# MEMS Mirrors for LiDAR: A Review

**DOI:** 10.3390/mi11050456

**Published:** 2020-04-27

**Authors:** Dingkang Wang, Connor Watkins, Huikai Xie

**Affiliations:** Department of Electrical and Computer Engineering, University of Florida, Gainesville, FL 32611, USA; noplaxochia@ufl.edu (D.W.); watkins.connor@ufl.edu (C.W.)

**Keywords:** LiDAR, optical scanner, laser scanning, mems mirror, micromirror

## Abstract

In recent years, Light Detection and Ranging (LiDAR) has been drawing extensive attention both in academia and industry because of the increasing demand for autonomous vehicles. LiDAR is believed to be the crucial sensor for autonomous driving and flying, as it can provide high-density point clouds with accurate three-dimensional information. This review presents an extensive overview of Microelectronechanical Systems (MEMS) scanning mirrors specifically for applications in LiDAR systems. MEMS mirror-based laser scanners have unrivalled advantages in terms of size, speed and cost over other types of laser scanners, making them ideal for LiDAR in a wide range of applications. A figure of merit (FoM) is defined for MEMS mirrors in LiDAR scanners in terms of aperture size, field of view (FoV) and resonant frequency. Various MEMS mirrors based on different actuation mechanisms are compared using the FoM. Finally, a preliminary assessment of off-the-shelf MEMS scanned LiDAR systems is given.

## 1. Introduction

LiDAR, or Light Detection and Ranging, is a dynamic distance measurement method. LiDAR was demonstrated in the early 1960s and it was first utilized in meteorology [1]. LiDAR soon found its uses in agricultural and archaeological surveys [2,3]. On the agricultural robots and agricultural airplanes, LiDAR can help classify plant species and analyze their growing states [3,4]. LiDAR is also very useful in archaeology. LiDAR can help to build a high-resolution dataset quickly and inexpensively. The LiDAR dataset can be easily integrated into a Geographic Information System (GIS) and can be used in municipal statics and planning, and the search for archeological sites. In recent years, LiDAR has become tremendously valuable in autonomous vehicles, including self-driving cars, automatic guided vehicles (AGVs), and unmanned aerial vehicles (UAVs). Autonomous vehicles can use LiDAR for obstacle detection and avoidance, object recognition and tracking, and simultaneous localization and mapping (SLAM) [5,6,7]. With the advancement of self-driving technologies, the demand for LiDAR is rapidly increasing.

**Figure 1:**
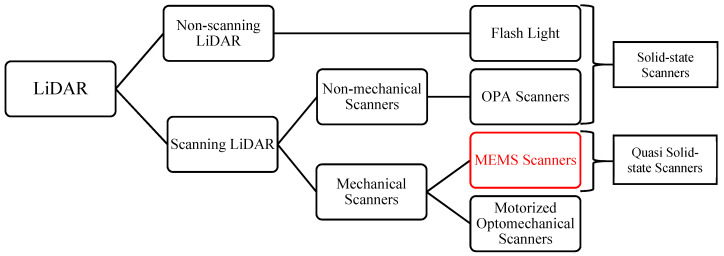
Different types of LiDAR scanners.

LiDAR uses a modulated laser as the carrier to measure the distance. A laser rangefinder can only measure the range in its instantaneous field of view (FoV). To create a 3D LiDAR point cloud with X, Y, and Z coordinate information, the laser must be delivered to all the directions in the desired FoV. LiDAR can be categorized according to how they scan the laser beam. As shown in Figure 1, there are two types of LiDAR: non-scanning LiDAR and scanning LiDAR. The most commonly used non-scanning LiDAR type is Flash LiDAR [8]. Among the scanning LiDAR, non-mechanical scanning LiDAR systems often use optical phased arrays (OPAs) to steer the laser beam without any moving parts [9]. Motorized optomechanical scanning LiDAR and MEMS scanning LiDAR both have moving parts in their scanner, and they are both called mechanical scanning LiDAR. Flash LiDAR and OPA LiDAR are collectively called solid state LiDAR as they do not have moving parts for laser scanning. MEMS LiDAR are called quasi-solid-state LiDAR as their moving parts steer only the laser beam in free space without moving any optical components. These LiDAR types are briefly reviewed below.

### 1.1. Non-Scanning LiDAR

**Figure 2:**
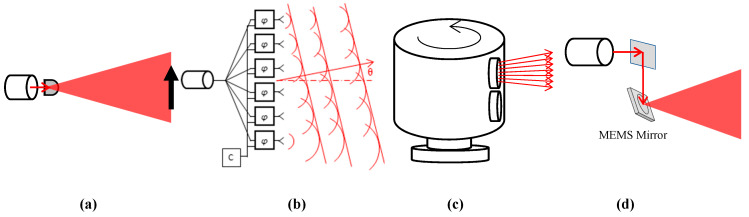
(**a**) A flash LiDAR with diffused light; (**b**) The principle of an optical phased array (OPA) scanner; (**c**) A LiDAR motorized spinning scanner; (**d**) A microelectronechanical systems (MEMS) laser scanner.

Non-scanning LiDAR is also called Flash LiDAR. “Flash” refers to the idea that the 2D FoV of interest is entirely illuminated by the laser source, like a camera with a flash light, while an array of photodetectors at the image plane simultaneously picks up the time-of-flight (ToF) information of individual pixels in the 2D FoV [10,11], as illustrated in Figure 2a. Flash LiDAR uses all solid-state components, which has the advantages of no moving parts, being resistant to vibrations, a compact size, and low price. Flood illumination implies that each pixel of the photodetector array receives only a small fraction of the returning laser power, leading to a low signal-to-noise ratio (SNR), which greatly limits the distance measurement range or demands very high laser power [8,12]. Furthermore, the resolution of the detector array-based non-scanning LiDAR is constrained by the size and density of the detectors array.

### 1.2. Non-Mechanical Scanning LiDAR

Scanning LiDAR systems steer the laser beam and they are more popular and more mature as they have an obvious advantage of a higher SNR compared to Flash LiDAR. There are two classes of laser beam scanning: non-mechanical scanning and mechanical scanning. The former is also called solid-state beam scanning because they have no moving parts [12]. Optical phased arrays (OPAs) are a typical solid-state beam steering technology that enable the non-mechanical steering of optical beams [13], as shown in Figure 2b. OPAs have the benefits of high stabilization, random-access pointing and good optical power handling capability. The laser power is split into an array of transmitters whose phases can individually controlled. By dynamically adjusting the relative phase shifts among the transmitters, a laser beam can be formed and steered [14]. The OPAs with several types of phase modulators have been reported, based on different approaches using liquid crystals, MEMS, or silicon photonics [9,15]. 

Liquid crystal OPA devices have the advantages of low driving voltage and simple implementation [16]. The maximum scanning angle is limited to around ±10° because of the low efficiency at a wider angle. The steering time is typically several milliseconds, which is not fast enough for many LiDAR scanners. MEMS-based OPAs are developed for higher efficiency, faster steering speed and no changes of polarization. With an array of 2-axis MEMS mirrors [17,18], both azimuth and elevation can be steered on one surface rather than being separated as is the case with reflective liquid crystal arrays. However, most of the MEMS-based OPAs still have moving parts, so they are not, strictly speaking, solid-state beam scanning. Silicon photonic phased arrays have the benefits of large-scale arrays, CMOS process compatibility, high integration and low cost [19]. S. Chung et al. developed a 1D silicon photonics-based OPA with 1024 elements, which is among the largest arrays reported up to now [20]. The fabrication was done with a standard 180 nm silicon-on-insulator (SOI) wafer process. A maximum scanning angle of 45° and an angular resolution of 0.03° were realized with thermal-optical phase modulation, and the response time was about 66 μs [21]. The big challenge now is how to increase the output optical power of silicon photonics phased arrays to the level for practical use [22,23,24].

Both non-scanning and non-mechanical scanning LiDAR are often referred as solid-state LiDAR.

### 1.3. Motorized Optomechanical Scanning LiDAR

Motorized optomechanical scanners are the most common type of LiDAR scanners [25]. In 2007, Velodyne released the first 64-line LiDAR based on a motorized scanner and a stack of multiple lasers and photodetectors, which has dominated the self-driving car market for a decade. This type of scanner can be developed with a long ranging distance, a wide FoV and a fast scanning speed [26]. There are several types of motorized optomechanical scanners. The most common one is built with multiple channels of transmitters and receivers stacked vertically and rotated by a motor to generate a full 360° FoV with multiple horizontal lines (Figure 2c) [27]. The signals and power may have to be wirelessly transmitted from the rotating part to the base board [25]. Such LiDAR are not power-efficient and are vulnerable to mechanical shock and wear [27]. In addition, their vertical resolution is fixed and dependent on the number of transmitter and receiver channels, so a high vertical resolution is always at the price of high cost. 

Furthermore, most of the motorized scanned LiDAR available on the market target geological surveys on airplanes or self-driving cars [25]. The high cost of such mechanical scanning LiDAR greatly obstructs the progress of autonomous vehicles. With the advancement of autonomous vehicles, especially unmanned aerial vehicles (UAVs), the market and the demand for compact LiDAR are quickly expanding. However, current LiDAR solutions based on motorized scanners either perform poorly, or are costly, bulky, and power-hungry. For instance, the challenge for self-driving cars is that LiDAR technology is expensive—currently, a LiDAR unit suitable for self-driving cars can cost up to USD 80,000, making it the most expensive element in a self-driving car.

### 1.4. MEMS Mirror-Based Quasi Solid-State LiDAR

As discussed above, both solid-state LiDAR and mechanical scanning LiDAR have major obstacles to overcome before they can be practically used in self-driving cars. Fortunately, Micro-Electro-Mechanical Systems (MEMS) technology provides a viable alternative. MEMS mirrors can steer, modulate, and switch light, as well as control phase. MEMS mirrors have already found enormous commercial success in projectors, displays, and fiber optic communications [28]. The most critical characteristics of MEMS mirrors lie in the fact that they are small and steer light in free space. Thus, compared to motorized scanners, MEMS scanners are superior in terms of size, scanning speed, and cost [29]. In the scheme of a MEMS mirror-based LiDAR, only the tiny mirror plate (whose diameter is in the range of 1–7 mm) of the MEMS device moves while the rest of the components in the system are stationary. Thus, MEMS LiDAR are often referred to as quasi-static-state LiDAR, an ultimate compromise of solid-state LiDAR and mechanical scanning LiDAR. 

LiDAR laser scanners have different requirements for different application scenarios, which brings both challenges and new opportunities for MEMS mirrors. Thus, the objective of this article is to review MEMS mirrors for LiDAR and MEMS mirror-enabled LiDAR systems. For MEMS mirrors for applications in other areas, please refer to ref. [30] for projection displays and medical imaging, and refs. [31] and [32] for MEMS-based endoscopic optical imaging.

There are several factors that affect the performance of a MEMS mirror. Thus, a Figure of Merit (FoM) that combines multiple critical factors is typically used to determine the suitability of various MEMS mirrors for a specific application. For instance, M. Saleem et al. used a FoM combining the deflection angle, power consumption, and actuator temperature to optimize their electrothermal MEMS mirror design for endoscopic optical coherence tomography (OCT) applications [33]. U. Baran et al. defined a FoM as the multiplication of the optical scanning angle, mirror width along the scanning direction, and resonant frequency of scanning mirrors [34] to compare piezoelectric MEMS mirrors for high-resolution displays. In this paper, a FoM is defined as the product of the scan angle, mirror area, and resonant frequency, and will be used to compare various MEMS mirrors for LiDAR applications.

### 1.5. The Scope and Organization of this Review Article

In addition to being applied in various LiDAR architectures, such as ToF LiDAR, structured light LiDAR, or Frequency-Modulated Continuous Wave (FMCW) LiDAR, all of the laser projection/scanning methods mentioned above can be used for generating structured light for 3D profiling such as 3D face recognition and 3D mapping [35,36,37]. This paper mainly focuses on MEMS scanners for LiDAR. Readers may use a similar methodology to analyze MEMS scanners for 3D profiling applications.

The review is organized as follows. First, LiDAR principle, LiDAR beam scanning metrics, and the FoM of MEMS LiDAR scanners will be introduced in Section 2. In Section 3, the MEMS LiDAR architectures based on 1D MEMS mirrors will be presented, followed by a review of 1D MEMS mirror-based LiDAR. In Section 4, the MEMS LiDAR architectures based on 2D MEMS mirrors will be described, followed by a review of 2D MEMS mirror-based LiDAR. In Section 5, a summary of the state of the art of the MEMS mirrors development as well as the outlook of MEMS LiDAR will be given. 

## 2. Laser Scanning Metrics for MEMS LiDAR

**Figure 3:**
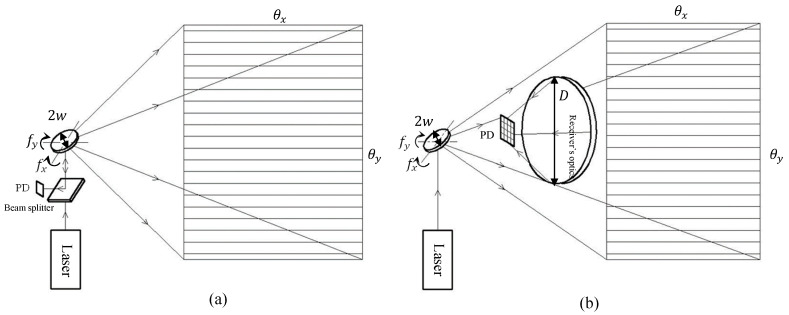
The schematics of MEMS scanned LiDAR with (**a**) coaxial architecture, and (**b**) non-coaxial architecture. *2w*: the dimension of the MEMS mirror plate. $f_{x},\;f_{y}$: the horizontal and vertical scanning frequency of the MEMS mirror. $\theta_{x}$ and $\theta_{y}:\;$ the horizontal and vertical optical scanning FoV. *D*: the dimension of the receiver’s optical aperture.

A simplified MEMS LiDAR is sketched in Figure 3, where a modulated laser is incident on a MEMS mirror that scans the laser beam to an object. The echoed laser signal from the object is then picked up by a photodetector and the time of flight can be used to extract the distance. The main scanning characteristics of the MEMS mirror include the scanning Field of View (FoV, or *θ*), scanning optical aperture (i.e., MEMS mirror size *2w*), scanning frequency (*f*), and scanning robustness (corresponding to the resonant frequency of the MEMS mirror, *f*_0_). These scanning metrics are discussed below, which are then combined to define an FoM. 

### 2.1. Scanning FoV

The FoV of the LiDAR is the scanning angular range, *θ,* of the laser scanner if the optical receiver has a sufficiently large acceptance FoV. Different applications have different requirements on FoV. In the case of autonomous driving, the FoV of interest for LiDAR is mainly in the forward direction that the vehicle heads to [38,39,40], which is similar to the cameras or radars systems used for driving assistance on cars today [37,41]. For example, a LiDAR with a horizontal FoV of 115° and a vertical FoV of 25° can be mounted at the front of a car for enhanced driving assistance. In the case of LiDARs for geological survey, the FoV of interested is in the downward direction [11,42]. For the near-field detection on self-driving cars, a LiDARs with a 180° × 180° fisheye FoV may be used to monitor the blind spot of a car [43]. Thus, the required FoV for a LiDAR may range anywhere from a few degrees to over 100 degrees.

### 2.2. Scanner’s Optical Aperture

The spatial resolution of LiDAR is determined by the divergent angle of the laser beam, which is given by [44],

(1) $$\theta_{min}≅\frac{M^{2}\lambda_{0}}{\pi w_{0}}$$

where $\theta_{min}$ is the half divergence angle of the laser beam, $\lambda_{0}$ is the wavelength of the laser, $w_{0}$ is the half beam waist of the laser (which is usually limited by the MEMS mirror dimension *w*) and *M*^2^ is the laser beam quality. An angular resolution of less than 1 mrad is typically required for LiDAR applications. This will require the scanning mirror plate to have a size of 1 mm for lasers with good beam quality and 3 mm for edge-emitting pulsed laser diodes with poor beam quality [45].

The maximum detectable distance of a LiDAR is determined by the minimum detectable power of the photodetector. The optical power detected by the photodetector is proportional to the receiver’s optical aperture, which is given by [46],

(2) $$P_{r}=P_{s}\eta_{t}\times \frac{\rho }{r^{2}}\times \frac{\pi D^{2}\eta_{r}}{2}$$

where:

*P_r_* = received signal power (W);
*P_s_* = source laser power (W);
*η_t_* = transmitter optical efficiency;
*ρ* = the reflectivity of the target object;
*r* = range from the transmitter to the target (m);
*D* = receiver aperture diameter (m);
*η_r_* = receiver optical efficiency.

### 2.3. Scanning Speed and Resonant Frequency

Both the fast axis scanning and the slow axis scanning are important for the LiDAR scanner. The frame rate equals the number of scanned frames in the slow scanning axis per the unit time. The resolution in pixels along the fast scanning axis (i.e., the horizontal axis in Figure 3), *p_h_*, is determined by the ToF measurement rate, *n*, and the fast-axis scanning frequency, *f_h_*, as expressed in Equation (3), and the resolution in the slow axis (i.e., the vertical axis in Figure 3), *p_v_*, is the ratio of *f_h_* and the slow-axis scan frequency, *f_v_*, as expressed in Equation (4). The fast-axis and slow-axis scanning frequency must be balanced for the trade-off of the resolution and frame rate. For a motorized LiDAR on a rotational stage, the fast-axis scanning is realized by quickly switching a laser array on and off in the vertical direction, and typically generating 16 to 128 vertical pixels, while the slow-axis scan frequency is the rotation rate of the motor, which typically ranges from 5 Hz to 30 Hz [25]. For MEMS scanned LiDAR for self-driving cars, the fast-axis (horizontal) scanning is in the range of 0.5–2 kHz in the horizontal direction, and the slow-axis (vertical) scanning is typically 10–30 Hz.

(3) $$p_{h}=\frac{n}{2f_{h}}$$

(4) $$p_{v}=\frac{f_{h}}{f_{v}}$$

A MEMS mirror with a high resonant frequency is preferred not only because it can scan fast or obtain high resolutions or high frame rates, but also because higher resonant frequency leads to greater robustness. The resonant frequency of a MEMS mirror is given by:

(5) $$f_{0}=\frac{1}{2\pi }\sqrt{\frac{k}{m}}$$

where *m* is the equivalent mass and *k* is the equivalent spring constant of the MEMS mirror. The quality factor (Q factor) of a resonator can be defined as the ratio of the resonator’s center frequency *f_0_* to its bandwidth Δf:

(6) $$Q=\frac{f_{0}}{\Delta f_{0}}$$

The quality factor can be used to estimate the maximum scanning angle at the resonant frequency, *θ_r_*, i.e.,

(7) $$\theta_{r}\approx Q\cdot \theta_{s}$$

where *θ_s_* is the non-resonant scanning angle. According to Equation (7), a MEMS mirror with a high Q-factor can have a large scanning angle at resonance, but according to Equation (6), the resultant bandwidth will be small, which means the scanning angle will be very sensitive to even a small change in the resonant frequency. Ideally, the MEMS mirror is expected to operate exactly at the resonant frequency and scan a constant angular range with a fixed phase lag [41]. However, any changes in the temperature, humidity or pressure in the surrounding environment or changes in the wear or stress relaxation of the MEMS actuation structures may affect the stiffness of the MEMS mirror and cause a shift of the resonant frequency. If there is no feedback control to detect the shift in the resonant frequency and adjust the working frequency immediately, a significant change in the scanning angle and phase delay are resulted. LiDAR are expected to operate in dynamically changing and harsh environments, so a real-time environment monitoring system or MEMS mirror position sensing and feedback control is required. 

Resonant scanning MEMS mirrors with low Q are less problematic because their bandwidths at resonance are larger and the phase slope is smoother. For instance, an electrothermal MEMS mirror with a resonant frequency of 1.89 kHz has a Q factor of 50 [47]; this mirror can generate stable scan without mirror position monitoring or feedback control. However, resonant scanning with low Q must have a large quasi-static scan angle. 

### 2.4. Scanner’s Size and Weight

The optical aperture of a laser scanner must be large for high-resolution scanning, but the overall size of the scanner must be small for a compact LiDAR. The commercially available motor scanned LiDARs are getting smaller. For example, the Velodyne Alpha Prime with 128 channels has a size of 141 × 165 × 165 mm^3^, and weighs only 3.5 kg [11]. Although the LiDAR size and weight are not critical for self-driving cars, robotics mobile platforms usually have stringent size and weight requirements. For example, one popular LiDAR for robotics applications, Hokuyo UST-10LX, weighs only 130 g [48], but this motorized LiDAR only has a 1D FoV. A lot of micro-robotics weigh under 10 g [49], so ultra-small LiDAR scanners are required. 

Using MEMS mirrors can greatly reduce the size and weight of LiDAR scanners. For example, a UAV-borne LiDAR with an electrostatic MEMS mirror only weighs ~45 g and can fit in a volume of 70 mm × 60 mm × 60 mm [50].

### 2.5. Typical Requirements for MEMS Mirrors for LiDAR Applications

As analyzed above, the baselines for MEMS mirrors for different applications are summarized in Table 1. LiDAR for self-driving cars are required to have good resolution over long detection distances, so a mirror size of at least 2 mm is required. Immunization to mechanical vibrations is also critical for automotive-grade products [51], so resonant frequencies higher than 0.8 kHz are typically required for MEMS mirrors for LiDAR on self-driving cars. The scanning angle of one MEMS mirror needs to be greater than 25° and a wider FoV can be achieved through the combination of multiple lasers and MEMS mirrors. The requirements under other applications are also given based on their working conditions. They are usually less critical than self-driving cars.

### 2.6. Figure of Merit

According to the above discussion, the scanning FoV, optical aperture and resonant frequency of a MEMS scanning mirror are the most critical parameters for LiDAR applications. So, a figure of merit (FoM) combining these parameters is defined to evaluate the performance of a MEMS mirror for LiDAR, i.e.,

(8) $$FoM=\theta_{e}\cdot d_{e}\cdot f_{e}$$

where *θ_e_* is the effective optical scanning angular field of view in radian, $d_{e}$ is the effective dimension of the mirror plate in mm, and *f_e_* is the effective resonant frequency of the MEMS mirror in kHz. Here $d_{e}$ is defined as follows,

(9) $$d_{e}=\sqrt{\frac{4A}{\pi }}$$

where *A* is the area of the mirror plate in mm^2^. For 1D scanning mirrors with a maximum resonant/non-resonant scanning angle of $\theta_{0}$ and resonant scanning frequency of $f_{0}$, *θ_e_* and *f_e_* are defined as follows,

(10) $$\theta_{e}=\theta_{0}/\beta_{1D}$$

(11) $$f_{e}=f_{0}$$

where $\beta_{1D}$ is a weight factor accounting for the Q-factor for 1D resonant scanning and is defined as,

(12) $$\beta_{1D}=\{\begin{array}{ll} \log Q & \;for\;resonant\;scanning\\ 1 & \;for\;non-resonant\;scanning \end{array}$$

where *Q* is the quality factor of the angular scanning mode of the mirror. For 2D scanning mirrors, *θ_e_* and *f_e_* are defined as,

(13) $$\theta_{e}=\frac{\sqrt{\theta_{0,x}\theta_{o,y}}}{\beta_{2D}}$$

(14) $$f_{e}=\sqrt{f_{0,x}f_{0,y}}$$

where *θ*_0,*x*_ and *θ*_0,*y*_ are the maximum scan angles in the x- and y-axis, respectively, *f*_0,*x*_ and *f*_0,*y*_ are the resonant frequencies in the x- and y-axis, respectively, and $\beta_{2D}$ is a weight factor accounting for the Q-factor for resonant scanning and is defined as,

(15) $$\beta_{2D}=\{\begin{array}{c} \log\left(Q_{x}Q_{y}\right)\;for\;double\;resonant\;scanning\\ \log Q\;\;\;\;\;\;\;\;\;for\;single\;resonant\;scanning\;\;\;\\ 1\;\;\;\;\;\;\;\;for\;non-resonant\;scanning.\; \end{array}$$

For MEMS mirrors with Q lower than 10, the Q is taken as 10 for simplicity. In general, MEMS mirrors with larger FoM are more suitable for LiDAR applications, and 2D MEMS mirrors are desired in most cases. However, 1D MEMS mirrors usually have simpler structures and can be more easily designed with a wide scan angle, large aperture and high frequency. A more detailed analysis will be given for 1D MEMS mirrors in Section 3 and 2D MEMS mirrors in Section 4. 

## 3. 1D MEMS Mirrors

In this section, we will first discuss the architecture of LiDAR with 1D MEMS mirrors, and then use the FoM defined in Equation (8) to compare various 1D MEMS mirrors already applied for LiDAR.

### 3.1. The Architectures of LiDAR with 1D MEMS Mirrors

**Figure 4:**
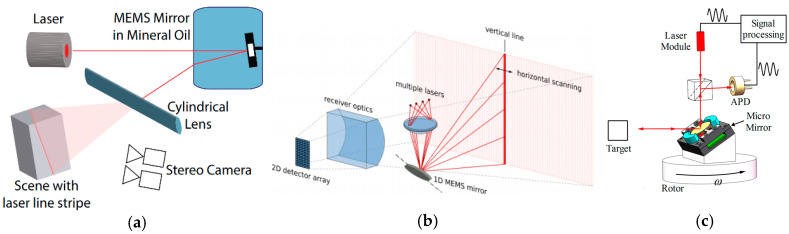
(**a**) The structured light camera design uses an 1D MEMS mirror and diffused laser [52]; (**b**) The LiDAR designed by Infineon uses a 1D MEMS mirror and a lasers array [54]; (**c**) The LiDAR with an 1D MEMS mirror and a motor for 2D scanning [56].

A 3D LiDAR can be built with a 1D MEMS mirror and a diffused laser beam, as shown in Figure 4a [52] and also found in [53]. The horizontally scanned beam from the MEMS mirror is diffused into a laser line by a diffuser lens [53]. Thus, the scanning beam can cover both the vertical and horizontal direction. The horizontal resolution in pixels is determined by the measurement sampling rate and the MEMS mirror scanning frequency. A photodetector (PD) 1D array is parallel to the diffused laser line and its number of PD elements determines the vertical resolution. High vertical resolution can be achieved at the cost of a large number of PD elements. Another common issue with this LiDAR is that the maximum detection distance is short. The first reason is that the power of the laser is distributed to a line, which results in a low optical power density. The second reason is that each PD element has an acceptance angle in the horizontal direction as wide as the scanning angle of the MEMS mirror, which sacrifices the SNR of the detected signal.

A similar architecture uses an array of laser sources to form the vertical scanning lines, a 1D MEMS mirror to scan the vertical lines in the horizontal direction, and a 2D PD array to collect the optical signals from the target [54], as shown in Figure 4b. This architecture can partially solve the problems of low laser power density and low SNR of the detected signal and take a step closer to realize commercial LiDAR for autonomous vehicles [55]. However, an extra alignment and assembly efforts are required to align multiple lasers to the MEMS mirror plate. A potential problem with laser collimation with such a small MEMS mirror may result in a poor angular resolution of the LiDAR.

In addition, mounting a 1D MEMS mirror on a motorized 1D scanner can create a 2D laser scanner for 3D LiDAR. One example is shown in Figure 4c. The MEMS mirror and the motorized scanner scan orthogonally to create a 2D scanning FoV. The motorized scanner can be a rotary motor [56]. The motorized scanners have the advantage of a wide scanning angle up to 360° for the slow axis scanning while the MEMS mirrors have the benefits of high resonant scanning frequency in kHz for the fast axis scanning. The optical receiver can be coaxial with the transmitted laser [56], which means the backscattered light goes through the MEMS mirror along the same path with the transmitted laser. The advantage of the coaxial architecture is that only one photodetector is needed, which greatly simplifies the structure of the LiDAR and the signal processing units. However, the maximum distance will be limited by the size of the MEMS mirror aperture according to Equation (2). The optical receiver may also be a separate detector with a photodetector array and a wide-angle optics. This structure may have a larger optical aperture for a longer detection distance at the cost of a large detector array. 

### 3.2. Resonant Scanning 1D MEMS Mirrors

**Figure 5:**
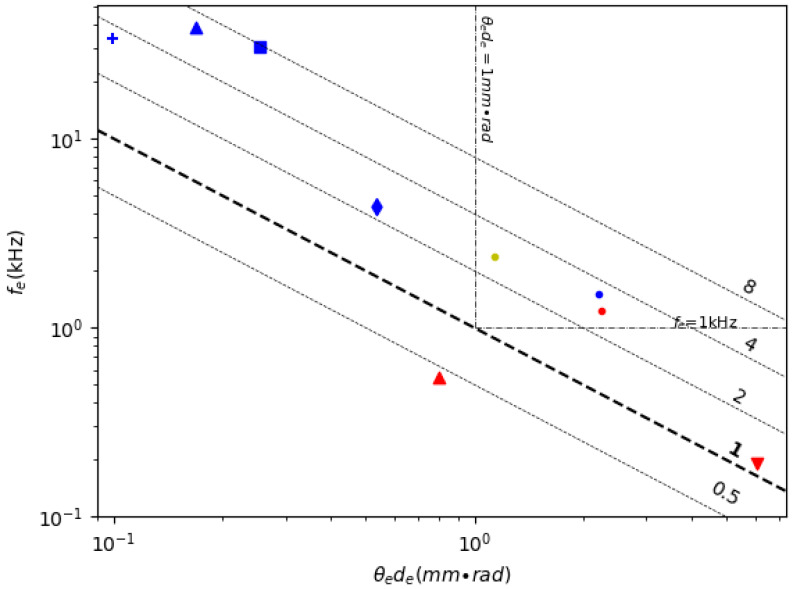
A comparison of 1D resonant scanning MEMS mirrors (The markers are listed in the table above. The same as below.).

Resonant scanning 1D MEMS mirrors have been widely used for making MEMS LiDAR due to their large angle and relatively simple structures. Table 2 lists the FoMs for various 1D resonant scanning MEMS mirrors reported in the literature; the FoMs are calculated using Equations (8)–(14). Figure 5 shows a scatter plot of the robustness (i.e., resonant frequency) versus the combined geometric scanning characteristics (i.e., *θ_e_d_e_*) plus the contour of the FoM values of the MEMS mirrors listed in Table 2. Any MEMS mirrors with FoM greater than 1 mm × rad × kHz may find some use in the large variety of LiDAR systems, but different applications may set different requirements. For instance, for MEMS mirrors used in a drone, in which vibrations are strong, the resonant frequency may need to be greater than 1 kHz. For a free moving robot that needs to see around, the mirror dimension-scan angle product may need to be at least 1 mm × rad. Two dashed lines are drawn on Figure 5, corresponding to the resonant frequency equal to 1 kHz and the mirror dimension-angle product equal to 1 mm × rad, respectively.

**Figure 6:**
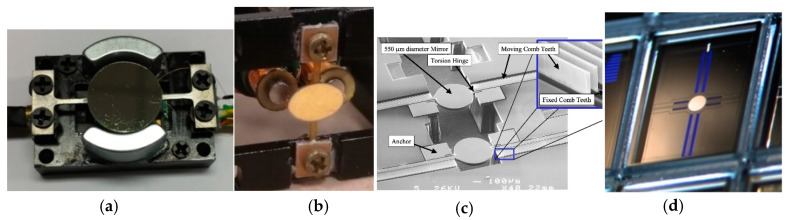
High FoM 1D resonant MEMS mirrors. (**a**) An electromagnetic MEMS mirror made with Ti-alloy structure material [56]; (**b**) An electromagnetic MEMS mirrors made with Flexible Printed Circuit Board (FPCB) structure material [62]; (**c**) An electrostatic MEMS mirror [64]; (**d**) An electrostatic MEMS mirror [60].

As can be seen in Figure 5, electromagnetic MEMS mirrors, shown in Figure 6a,b, have high FoM over 1. The mirror shown in Figure 6a has a large mirror size (12 mm in diameter) and wide angle (26°), as well as a relatively high resonant frequency (1.24 kHz) at the same time [56]. This 1D resonant scanning electromagnetic MEMS mirror uses a 1D position sensitive detector (PSD) to monitor the scanning angle and provides feedback to the mirror controller, which increases the size and cost of the scanner and LiDAR system [56]. Integrated angular sensing elements can be fabricated directly with MEMS mirrors, such as inductive sensing [57], piezoresistive sensing [58], or Hall sensing [59]. An electrostatic MEMS mirror with a high FoM of 7.79 is shown in Figure 6b [60], but these high-FoM electrostatic MEMS mirrors usually have small mirror plates. A high-FoM electrothermal MEMS mirror with an optical scan angle as large as 170° is shown in Figure 6c [61]. 

### 3.3. Non-Resonant Scanning 1D MEMS Mirror

**Figure 7:**
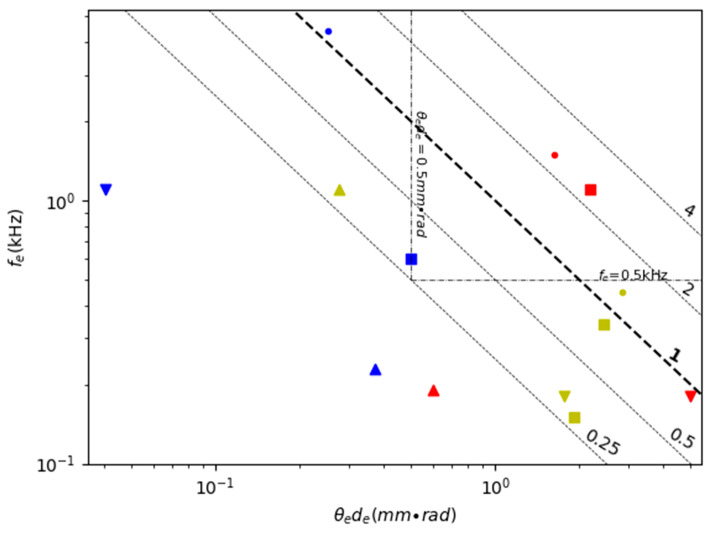
A comparison of 1D non-resonant scanning MEMS mirrors.

**Figure 8:**
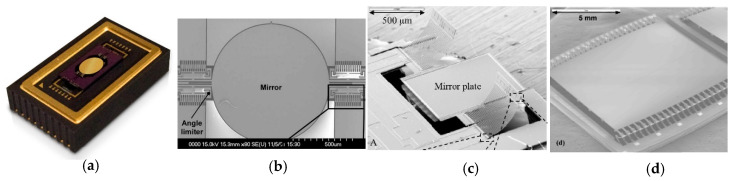
High FoM 1D non-resonant MEMS mirrors. (**a**) An electromagnetic actuator [67] (Image courtesy of Maradin, Yokneam, Israel); (**b**) An electrostatic MEMS mirror [68]; (**c**) An electrothermal MEMS mirror with a 180° non-resonant optical scanning angle [69]; (**d**) An electrothermal MEMS mirror with a mirror size of 10 × 10 mm^2^ [70].

Table 3 lists the FoMs of various 1D non-resonant scanning MEMS mirrors. Figure 7 shows a scatter plot of the robustness (i.e., resonant frequency) versus the combined geometric scanning characteristics (i.e., *θ_e_d_e_*) plus the contour of the FoM values of the MEMS mirrors listed in Table 3. The FoMs of 1D non-resonant MEMS mirrors are generally lower than those of resonant mirrors, most of which are less than 1. Among these 1D non-resonant scanning MEMS mirrors, the electromagnetic actuation mirror type shows relatively higher FoM values than the two other actuation types [67] (Figure 8a). One electrostatic MEMS mirror achieved an FoM over 1 because of its large quasi-static scan angle [66], and a typical electrostatic MEMS mirror design is shown in Figure 8b [68]. One electrothermal MEMS mirror (Figure 8c) achieved an FoM of 1.28 because of its extra large quasi-static scan angle of 180° [69], but the mirror plate size is only 0.8 × 0.8 mm^2^. Another electrothermal MEMS mirror has an extra large mirror size of 10 × 10 mm^2^ [70].

One obvious advantage of non-resonant scanning MEMS mirrors is that they do not have the issues of significant changes in scanning angle and phase lag with the environmental fluctuations, so non-resonant scanning is more robust against the ambient temperature variations even without the mirror position feedback control. 

## 4. 2D MEMS Mirrors for LiDAR

This section will first discuss the architecture of LiDAR systems based on 2D MEMS mirrors. Similar to 1D MEMS mirrors, 2D MEMS mirrors can scan either axis in a resonant mode or non-resonant mode. Thus, there exist three scanning modes: resonant scans in both axes, resonant scan in one axis/non-resonant scan in the other axis, and non-resonant scans in both axes. Section 4.2, Section 4.3 and Section 4.4 will discuss 2D MEMS mirrors working in those three scanning modes in detail. 

### 4.1. Architecture of LiDAR with 2D MEMS Mirrors

**Figure 9:**
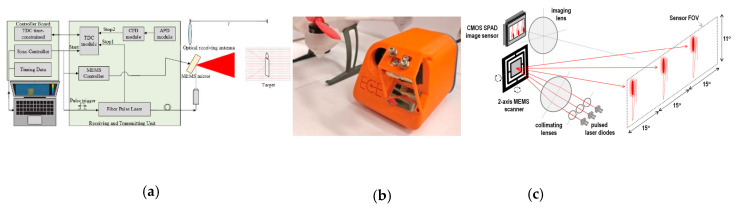
2D MEMS LiDAR systems built with (**a**) an electrostatic MEMS mirror and a single pixel receiver [74] (**b**) an electrothermal MEMS mirrors with a single pixel receiver [75]; (**c**) an electromagnetic MEMS mirror with a detector array [76].

LiDARs with 2D scanning MEMS mirrors always use collimated laser sources. The laser beam incident on the MEMS mirror with a similar size as the MEMS mirror, as shown in Figure 9a [74] and Figure 9b [75]. With multiple lasers with different incident angles, the overall scanning FoV can be multiplied. For example, Figure 9c illustrates a LiDAR system that consists of one MEMS mirror with a 15° × 11° optical scanning angle and three lasers with a 15° separation in the horizontal direction to create an overall FoV of 45° × 11° [76]. 

The optical receiver is usually a photodetector, or a photodetector array, with wide-angle optics to capture the overall MEMS scanning FoV. With a photodetector array, as the one shown in Figure 9c, each pixel only collects the light from a narrow FoV, so the SNR is enhanced. A single-pixel photodetector can also be used with a trade-off of the receiver’s optical aperture and acceptance angle. For example, a bare photodetector (Figure 9a) or a photodetector placed away from the image plane of the optical collection optics [75], was implemented in the LiDAR systems. An optical bandpass filter that matches the laser wavelength and the transmission angle is also needed to filter out the background light.

With additional optics, a wider scanning FoV can be achieved with 2D MEMS mirrors. For example, a fish-eye wide-angle optical lens is used to expand the scanning angle of a MEMS mirror from 28° to 45° [77]. Full 360° omnidirectional scanning has been demonstrated with 2D MEMS mirrors and omnidirectional optics [78,79]. 

### 4.2. Double Resonant Scanning

**Figure 10:**
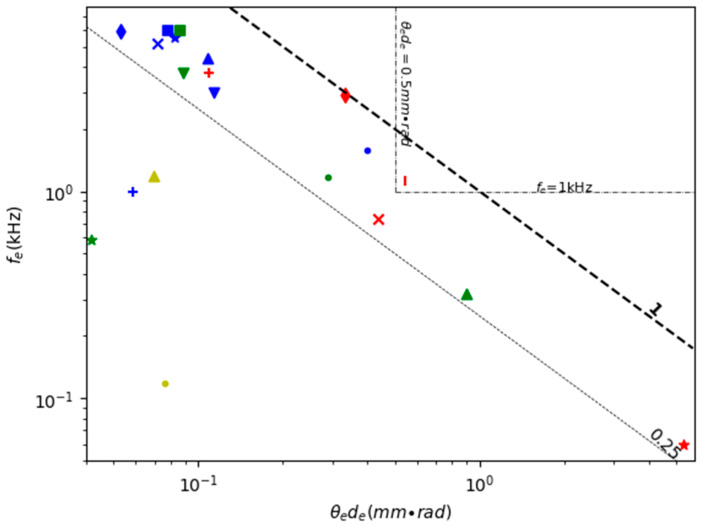
A comparison of 2D double resonant scanning MEMS mirrors.

Table 4 shows the comparison of the FoM of various 2D double resonant scanning MEMS mirrors. Figure 10 is a scatter plot of the robustness (i.e., resonant frequency) versus the combined geometric scanning characteristics (i.e., *θ_e_d_e_*) plus the contour of the FoM values of the MEMS mirrors listed in Table 4. Only a few of the list MEMS mirrors have FoMs close to 1. 

**Figure 11:**
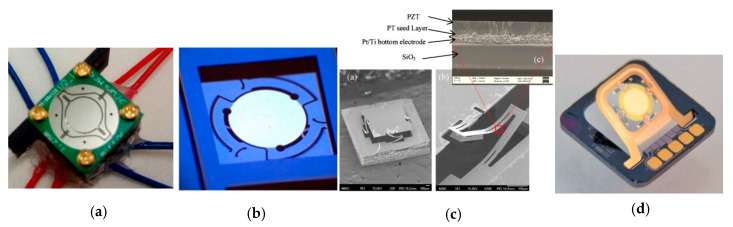
High FoM double resonant scanning MEMS mirrors. (**a**) Electromagnetic actuation [81]; (**b**) Electrostatic actuation [78]; (**c**) Piezoelectric actuation [95] (**d**) Electrothermal actuation [90].

For double resonant scanning MEMS mirrors, some of the electromagnetic MEMS mirrors show high FoM values larger than 0.5. A typical electromagnetic MEMS mirror design is shown in Figure 11a [81]. Some electrostatic MEMS mirrors also demonstrate FoM around 0.5 [78,85]. For example, the 7-mm electrostatic resonant scanning mirror shown in Figure 11b for omnidirectional scanned LiDAR [78] has a FoM of 0.62. Dual-resonant scanning piezoelectric MEMS mirrors also become an option for LiDAR scanners. A typical piezoelectric MEMS mirror is shown in Figure 11c [95]. Another piezoelectric MEMS mirror has a high FoM of 0.51 [87], which has a high *f_e_* of 6 kHz. Electrothermal MEMS mirrors are rarely used as double resonant scanning mirrors, with one example shown in Figure 11d [90].

Double resonant scanning generates Lissajous scanning patterns, which has been applied in compact laser scanning applications such as endomicroscopy [90] or projection display [96]. The x-axis and y-axis resonances of a 2D MEMS mirror can be designed at different frequencies so that the two scanning axes can be actuated at the respective resonant frequencies. In this double resonant scanning case, the frame rate and resolution are defined differently from Equations (3) and (4) [96]. Double resonant scanning by scanning both axes at their center resonant frequencies has issues of low frame rate and poor coverage (i.e., low spatial resolution). One solution to that is to shift the scanning frequencies slightly off the resonance peaks but still within the resonance bandwidths to balance the scanning frame rate, resolution and coverage [96]. However, this requires the MEMS mirror to have a wide resonance bandwidth (300 Hz for [96]), but that means the Q is low and thus the angular scanning range will be small. Due to process and packaging variations, the resonance frequency and Q factor of the same MEMS design will vary considerably from one device to another, posing big challenges to system calibration.

### 4.3. Double Non-Resonant Scanning

**Figure 12:**
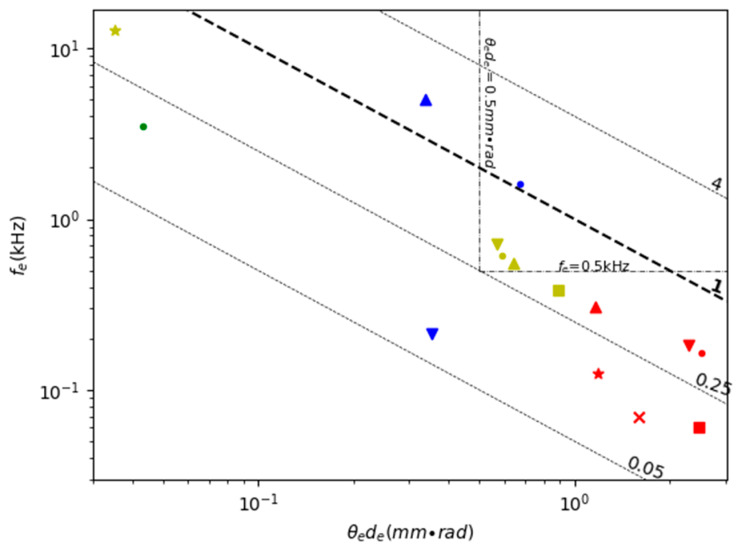
A comparison of 2D non-resonant scanning MEMS mirrors.

Table 5 shows the comparison of the FoM of various 2D non-resonant scanning MEMS mirrors. Figure 12 is a scatter plot of *f_e_* versus *θ_e_d_e_* plus the contour of the FoM values of the MEMS mirrors listed in Table 5. Double non-resonant scanning requires both directions to have wide non-resonant scanning angles. However, the resonant frequency is still taken into account in the FoM in Table 5 and Figure 12 because the resonant frequency largely determines the robustness of the mirror.

The values of the FoM for double non-resonant scanning MEMS mirrors are generally smaller than their double resonant scanning counterparts because both the values of the resonant frequency and the non-resonant scanning angle are smaller. There are only very few electromagnetic MEMS mirrors [97,103] and electrostatic MEMS mirrors [102] that have a resonant frequency over 1 kHz and FoM close to or above 0.5. However, double quasi-static scanning has the benefits of high scanning flexibility, adaptive FoV, and simple linear control, so it is more desirable for the LiDAR for robotics applications.

**Figure 13:**
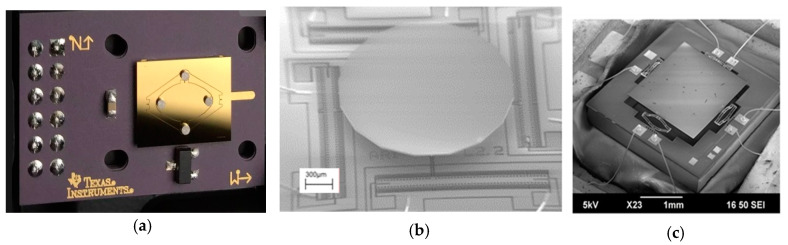
High FoM double non-resonant scanning MEMS mirrors. (**a**) Electromagnetic actuation [100] with compliant membrane (Image courtesy of Texas Instruments, Dallas, TX, USA); (**b**)Electrostatic actuation [102]; (**c**) Electrothermal actuation [106].

Electromagnetic actuation has the advantage of large force and can support large mirror size. However, electromagnetic MEMS mirrors usually require torsional beams or compliant membranes to guide the scanning mirror directions [97,98], which may limit the maximum non-resonant scanning angle. Large magnets or coils as well as relatively high power are needed for generating large electromagnetic force, which unfortunately reduces the resonant frequency and may make the mirror unstable under external vibration. For example, one electromagnetic MEMS mirror shown in Figure 13(a) developed by TI had a mirror size of 9 mm^2^ and could generate a 20° scanning angle but its resonant frequency was only 0.13 kHz [100]. According to Figure 12, electromagnetic MEMS mirrors generally have high *θ_e_d_e_* (above 1 mm × rad) but low resonant frequency (under 0.3 kHz).

Similar trade-offs exist in electrostatically actuated MEMS mirrors and electrothermally actuated MEMS mirrors. Gimbal-less structures are often used for non-resonant scanning MEMS mirrors. Gimbal-less MEMS mirrors typically have four groups of actuators with similar structures arranged on the four sides of the mirror plate for large actuation range. One electrostatic mirror developed by Mirrocle, shown in Figure 13b, also added transformer and lever structures to amplify the actuation range [102]. Electrothermal bimorph actuators have the advantages of large non-resonant scanning angle and high fill factor; they can use long electrothermal bimorph cantilevers or connect several electrothermal bimorph cantilevers in series to achieve large displacements and wide scanning angle [104,105,106,109]. One typical example of non-resonant scanning electrothermal MEMS mirrors designed for LiDAR is shown in Figure 13c [106]. Despite the advantages of low power consumption and high actuation speed, piezoelectric actuated mirrors are limited in their non-resonant scanning range. For example, the non-resonant scanning angle of this piezoelectric MEMS mirror is only 2.1° by 1.8°, with a low FoM of 0.15 [108].

Non-resonant scanning MEMS mirrors can also scan at high frequencies in the range of a few hundred Hertz. The scanning frequency bandwidth is usually limited by the drag-air damping and the resonant frequency. To improve the working frequency range of the non-resonant scanning, a closed-loop controller may be used. For instance, a 2 mm electrostatic MEMS mirror’s working bandwidth was extended from 600 Hz to 3 kHz by using a close-loop controller [110]. For electrothermal MEMS mirrors, the scanning frequency bandwidth is typically limited to a few hundred Hertz because of the need for thermal dissipation.

### 4.4. Non-Resonant Plus Resonant Scanning

**Figure 14:**
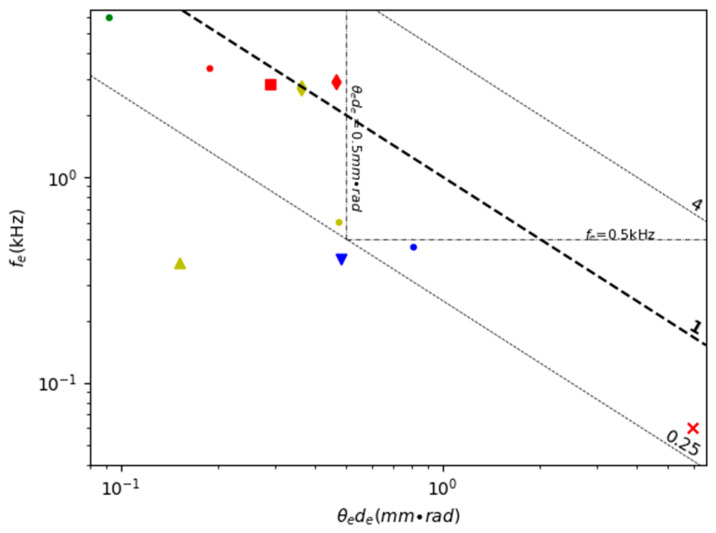
A comparison of 2D resonant plus non-resonant scanning MEMS mirrors.

Table 6 lists the MEMS mirrors with one non-resonant scanning and one resonant scanning. Figure 14 is a scatter plot of *f_e_* versus *θ_e_d_e_* plus the contour of the FoM values of the MEMS mirrors listed in Table 6. These 2D MEMS mirrors can scan one axis in a non-resonant mode at a relatively low frequency and scan the other axis at the resonant frequency, resulting in a raster scanning trajectory. The raster scanning trajectory provides a convenient means for LiDAR systems to generate real-time 3D point-clouds. The frame rate of the raster scanning is determined by the non-resonant scanning frequency, which typically ranges from 5 Hz to 100 Hz. According to Equation (4), the spatial resolution of the scanning is determined by the ratio between the resonant scanning frequency and the non-resonant scanning frequency. Thus, the scanning frame rate and resolution can be easily tuned by changing the non-resonant scanning frequency. The FoV along the slow scanning axis can also be tuned by changing the non-resonant scanning angle. A triangular actuation signal rather than a sinusoidal signal for the non-resonant scanning is preferred for a uniform scanning trajectory.

**Figure 15:**
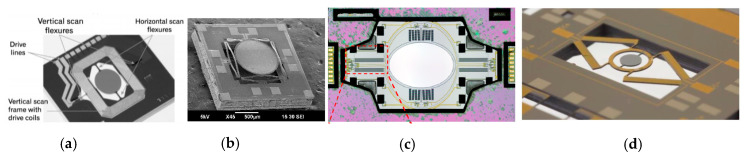
High-FoM 2D quasi-static and resonant scanning MEMS mirrors with (**a**) electromagnetic actuators [80]; (**b**) electrothermal actuators [109]; (**c**) electrostatic actuators [112]; (**d**) piezoelectric actuators [87].

As can be seen in Table 6, the MEMS mirrors with high FoM are mainly electromagnetic MEMS mirrors [80,111] and one electrothermal mirror [109]. Electromagnetic MEMS mirrors (one example is shown in Figure 15a) have the advantage of achieving large apertures and wide angles at the same time [80,111]. The resonant frequency of the fast axis of one electromagnetic MEMS mirror was 18 kHz—achieved by turning the stiffness of the torsional beam [111]. Electrothermal MEMS mirrors (one example is shown in Figure 15b) have the advantages of large non-resonant scan angle and high fill factor [109]; their resonant frequencies are mostly are in the range of 0.4–3 kHz [104,105,106,107,109]. Both electrostatic mirrors [112] and piezoelectric mirrors [87] can achieve high resonant frequencies, but their quasi-static scanning angles are relatively small (Figure 15c,d).

## 5. Summary and Outlook

Both 1D and 2D MEMS mirrors based on electrostatic (ES), electromagnetic (EM), electrothermal (ET) and piezoelectric (PE) actuation are analyzed in Section 3 and Section 4. Those MEMS mirrors are categorized into three working modes, i.e., resonance, non-resonance, or a combination of resonance and non-resonance scanning. Now let us revisit the minimum requirements for MEMS mirrors with various applications listed in Table 2, Table 3, Table 4, Table 5 and Table 6 and calculate their corresponding FoM values that are present in Table 7. As shown in Table 7, the minimum FoM ranges from 0.1 to 1.0 for various applications. Also tabulated in Table 7 are the statistics of the numbers of ES, EM, ET and PE MEMS mirrors for each application in terms of the FoM criteria. These data show that more than half of those MEMS mirrors can find potential use in one or more applications.

Overall, 1D scanning MEMS mirrors, including non-resonant scanning 1D mirrors and resonant scanning 1D mirrors, are more mature and usually have wider scanning angles, larger apertures and higher resonant frequencies, making them a good choice for LiDAR. Some 1D electromagnetic and electrostatic MEMS mirrors seem especially suitable for most LiDAR applications. However, to acquire 3D point clouds with 1D MEMS mirrors, either large detector arrays or motorized stages are needed, which complicates the LiDAR system. Thus, 2D MEMS mirrors are preferred for high-performance LiDAR. In general, there are multiple actuation mechanisms as well as multiple scanning modes to choose from. Deciding which combination of actuation mechanism and scanning mode to pick depends on the application. The FoMs shown in Table 2, Table 3, Table 4 and Table 5 and Figure 5, Figure 7, Figure 10, Figure 12 and Figure 14 can be used as a reference and provide a guide. Many electromagnetic and electrothermal MEMS mirrors meet the standard requirements for ground robotics, gesture recognition, and MAV.

In the last few years, the demand for LiDAR systems with greater scanning angles, speeds, and distances has led to the rapid development of a variety of MEMS mirrors. Some MEMS-scanned LiDAR products have already come to the market with test samples available for evaluation, and most of these products are targeted towards self-driving cars or ADAS applications. MEMS-scanned LiDAR products are gradually approaching the performance level of motorized LiDAR, but at a much lower price. The future of MEMS-scanned LiDAR is very promising.

## Figures and Tables

**Table 1:** The minimal requirements for MEMS mirrors for different LiDAR applications.

Applications	FOV (°)	Mirror Size (mm)	Resonant Frequency (kHz)
Self-Driving Cars	25	2	0.8
Blind-Spot Detection	120	1	0.5
Gesture Recognition	50	0.5	0.2
Ground Robotics	25	1	0.2
Micro Air Vehicles (MAVs)	30	1	0.4

**Table 2:** The FoMs of 1D resonant scanning MEMS mirrors.

Marker	Actuation Method	FoM	Mirror Plate Dimensions	Resonant Angle *θ*_0_		Resonant Frequency *f*_0_ (kHz)	Q	Ref.
				(°)	(rad)			
•	EM	2.79	D = 12 mm	26	0.45	1.24	253	[56]
▼	EM	1.16	28.2 mm^2^	62	1.08	0.19	12	[62]
▲	EM	0.44	D = 2 mm	30	0.52	0.55	20 *	[63]
■	ES	7.79	D = 0.8 mm	80	1.40	30.8	26800	[60]
▲	ES	6.48	D = 1.0 mm	45	0.79	38.5	49300	[60]
+	ES	3.38	D = 0.55 mm	25	0.44	34	273	[64]
•	ES	3.33	Ellipsoid, 2 mm × 4 mm^2^	180	3.14	1.5	10,000 *	[65]
♦	ES	2.36	D = 0.8 mm	58	1.01	4.4	50	[66]
•	ET	2.72	0.7 × 0.32 mm^2^	170	2.97	2.4	25	[61]

* The Q are estimated from similar designs. EM: Electromagnetic. ES: Electrostatic. ET: Electrothermal. PE: Piezoelectric. D: Diameter.

**Table 3:** The FoMs of 1D non-resonant scanning MEMS mirrors.

Marker	Actuation Method	FoM	Mirror Plate Dimension	Non-Resonant Scanning Angle *θ*		Resonant Frequency *f*_0_ (kHz)	Ref.
				(°)	(rad)		
•	EM	2.44	3.6 × 4.7 mm^2^	20	0.35	1.5	[67]
■	EM	2.40	3.6 × 8.5 mm^2^	20	0.35	1.1	[67]
▼	EM	0.89	D = 14.2 mm	20	0.35	0.18	[71]
▲	EM	0.11	28.5 mm^2^	6	0.10	0.19	[62]
•	ES	1.11	0.8 × 0.8 mm^2^	16	0.28	4.4	[66]
■	ES	0.30	D = 1 mm	28.8	0.50	0.6	[68]
▲	ES	0.09	1 × 1 mm^2^	18.8	0.33	0.23	[47]
▼	ES	0.04	0.96 × 0.11 mm^2^	6.5	0.11	1.1	[72]
•	ET	1.28	0.8 × 0.8 mm^2^	180	3.14	0.45	[69]
★	ET	0.83	1 × 1 mm^2^	124	2.16	0.34	[73]
▼	ET	0.32	6 × 6 mm^2^	15	0.26	0.18	[70]
▲	ET	0.31	0.7 × 0.32 mm^2^	30	0.52	1.1	[61]
■	ET	0.29	10 × 10 mm^2^	10	0.17	0.15	[70]

**Table 4:** The FoM of 2D double resonant scanning MEMS mirrors.

Marker	Actuation Method	FoM	Mirror Plate Dimension	Resonant Angle *θ_h_*		Resonant Angle *θ_v_*		*f_h_* (kHz)	*f_v_* (kHz)	*β*	Ref.
				(°)	(rad)	(°)	(rad)				
♦	EM	0.97	D = 1.5 mm	65	1.13	53	0.92	0.4	21.3	4.60 *	[80]
|	EM	0.61	D = 6.5 mm	18	0.31	30	0.53	0.674	1.87	4.88	[81]
+	EM	0.41	D = 1 mm	28	0.48	40	0.70	0.56	25	5.29	[82]
×	EM	0.32	2.6 × 3.6 mm^2^	26	0.45	24	0.42	1.4	0.39	3.45	[83]
★	EM	0.32	8 × 8 mm^2^	90	1.57	50	0.87	0.06	0.06	2.00	[84]
•	ES	0.62	D = 7 mm	26	0.45	26	0.45	1.57	1.57	8.01	[78]
▲	ES	0.48	D = 1 mm	44	0.77	24	0.42	26	1.4	7.00 *	[85]
■	ES	0.47	D = 1 mm	60	1.05	70	1.22	17.8	0.5	7.30 *	[60]
★	ES	0.46	1 × 1.1 mm^2^	45	0.79	30	0.52	10.3	1.9	7.30 *	[86]
×	ES	0.37	D = 1 mm	40	0.70	30	0.52	22	1.4	7.30	[85]
▼	ES	0.34	D = 1 mm	30	0.52	30	0.52	18	1.5	9.94	[87]
♦	ES	0.31	0.2 × 0.2 mm^2^	27	0.47	27	0.47	5.9	5.9	2.00	[88]
+	ES	0.06	D = 1 mm	18	0.31	10	0.17	1	1	4.00 *	[89]
▲	ET	0.08	D = 1 mm	16	0.28	10	0.17	1.19	1.18	3.18	[90]
•	ET+EM	0.01	2 × 1 mm^2^	10	0.17	3	0.05	0.2	0.07	2.00 *	[91]
■	PE	0.51	D = 1 mm	21	0.37	31	0.55	23.9	1.5	5.26 *	[87]
•	PE	0.34	2 × 2 mm^2^	28	0.48	40	0.70	25	0.56	5.60	[92]
▼	PE	0.33	1 × 1 mm^2^	42	0.73	40	0.70	1.46	0.95	7.40	[93]
▲	PE	0.28	4 × 7.4 mm^2^, corner shape	26	0.45	23	0.40	0.46	0.22	2.93	[94]
★	PE	0.02	1.1 × 1.1 mm^2^	5	0.09	5	0.09	0.58	0.58	2.60	[95]

* The Q are estimated from similar designs.

**Table 5:** The FoM of 2D double non-resonant scanning MEMS mirrors.

Marker	Actuation Method	FoM	Mirror Plate Dimension A	Non-Resonant Angle *θ_h_*		Non-Resonant Angle *θ_v_*		*f_h_* (kHz)	*f_v_* (kHz)	Ref.
				(°)	(rad)	(°)	(rad)			
■	EM	0.42	D = 2.5 mm	60	1.05	46	0.80	0.16	0.21	[97]
•	EM	0.42	4 × 4 mm^2^	32	0.56	32	0.56	0.16	0.17	[98]
▼	EM	0.35	4.2 × 3.2 mm^2^	16	0.28	16	0.28	0.24	0.39	[99]
▲	EM	0.15	8 × 8 mm^2^	15.7	0.27	16.2	0.28	0.06	0.06	[84]
★	EM	0.15	9 mm^2^	20	0.35	20	0.35	0.13	0.12	[100]
×	EM	0.11	D = 0.38 mm	240	4.19	240	4.19	0.07	0.07	[101]
★	ES	1.29	D = 0.8 mm	24	0.42	24	0.42	3.8	3.9	[102]
▼	ES	1.08	D = 1.6 mm	24	0.42	24	0.42	0.67	1.6	[102]
▲	ES	0.07	D = 1 mm	24.8	0.43	16.4	0.29	0.35	0.32	[103]
★	ET	0.45	D = 0.5 mm	4	0.07	4	0.07	12.8	12.8	[104]
■	ET	0.41	0.72 × 0.72 mm^2^	40	0.70	40	0.70	0.69	0.74	[105]
•	ET	0.36	2.5 × 2 mm^2^	15	0.26	12	0.21	0.7	0.53	[106]
▲	ET	**0.35**	0.9 × 0.9 mm^2^	36	0.63	36	0.63	0.55	0.55	[107]
♦	ET	**0.34**	0.5 × 0.5 mm^2^	102	1.78	79	1.38	0.17	0.87	[105]
•	PE	**0.15**	1.1 × 1.1 mm^2^	2.1	0.04	1.8	0.03	0.04	3.5	[108]

**Table 6:** Non-resonant plus resonant scanning 2D MEMS mirrors.

Marker	Actuation Method	FoM	Mirror Plate Dimension A	Resonant Angle *θ_h_*		Non-Resonant Angle *θ_v_*		*f_h_* (kHz)	*f_v_* (kHz)	*β*	Ref.
				(°)	(rad)	(°)	(rad)				
♦	EM	1.35	D = 1.5 mm	53	0.92	65	1.13	0.4	21.3	3.3 *	[80]
■	EM	0.82	D = 1.4 mm	43.2	0.75	24.3	0.42	18	0.44	2.7 *	[111]
•	EM	0.64	D = 1 mm	50	0.87	30	0.52	14.4	0.8	3.1 *	[82]
×	EM	0.36	8 × 8 mm^2^	90	1.57	16.2	0.28	0.06	0.06	1.0	[84]
•	ES	0.37	2.6 × 3.6 mm^2^	80	1.40	20	0.35	1.6	0.13	3.0 *	[112]
▼	ES	0.19	5 × 7.1 mm^2^	21.4	0.37	2	0.03	0.61	0.26	1.3	[113]
♦	ET	0.98	D = 1 mm	16	0.28	53	0.92	2.7	2.7	1.4	[109]
•	ET	0.29	2.5 × 2 mm^2^	20	0.35	15	0.26	0.7	0.53	1.6	[106]
▼	ET	0.13	D = 0.5 mm	1.6	0.03	4	0.07	12.8	12.8	2.2	[104]
▲	ET	0.06	0.5 × 0.5 mm^2^	50	0.87	14	0.24	0.17	0.87	1.7 *	[105]
•	PE	0.55	D = 1 mm	21.4	0.37	13.7	0.24	23.9	1.5	3.3	[87]

* The Q are estimated from similar designs.

**Table 7:** The minimum requirements and FoMs for MEMS mirrors for different LiDAR applications.

Applications	Baselines Requirements					The Number of MEMS Mirrors Meet FoM Requirement	
	$\theta_{e}\;({}^{\circ})$	$\theta_{e}\;(rad)$	$d_{e}\;(\mathrm{mm})$	$f_{e}\;(\mathrm{kHz})$	FoM (mm × rad × kHz)	1D	2D
**Self-Driving Cars**	25	0.44	2	0.8	0.7	EM: 5	EM: 3
						ES: 6	ES: 2
						PE: 0	PE: 0
						ET: 3	ET: 1
**Blind-Spot Detection**	120	2.09	1	0.5	1.0	EM: 4	EM: 1
						ES: 6	ES: 2
						PE: 0	PE: 0
						ET: 2	ET: 0
**Gesture Recognition**	50	0.87	0.5	0.2	0.1	EM: 7 ES: 7 PE: 0 ET: 6	EM: 15 ES: 11 PE: 6 ET: 8
**Ground Robotics**	25	0.44	1	0.2	0.1		
**Micro Air Vehicles (MAVs)**	30	0.52	1	0.4	0.2	EM: 6	EM: 12
						ES: 7	ES: 10
						PE: 0	ET: 7
						ET: 6	PE: 5
